# Relationship between bibliometric indicators and university ranking positions

**DOI:** 10.1038/s41598-023-35306-1

**Published:** 2023-08-30

**Authors:** Péter Szluka, Edit Csajbók, Balázs Győrffy

**Affiliations:** 1 Central Library, Semmelweis University, 1088 Budapest, Hungary; 2 Research Center for Natural Sciences, Institute of Enzymology, Magyar Tudósok Körútja 2, 1117 Budapest, Hungary; 3 Department of Bioinformatics, Semmelweis University, Tűzoltó Utca 7-9, 1094 Budapest, Hungary; 4 2nd Department of Pediatrics, Semmelweis University, 1094 Budapest, Hungary

**Keywords:** Information technology, Scientific data

## Abstract

A growing interest for demonstrating prestige and status of higher education institutions has spurred the establishment of several international ranking systems. A major percentage of these rankings include parameters related to scientific productivity. Here, we examined the differences between diverse rankings as well as correlation with bibliometric parameters and disciplines for the top universities. We investigated the top 300 universities from four international rankings, the Times Higher Education World University Ranking (THE), the QS World University Rankings (QS) the ShanghaiRanking-Academic Ranking of World Universities (ARWU) and the U.S.News Best Global Universities Ranking (USNews). The assessed parameters include ranking positions, size related and bibliometrics-related indicators of each selected ranking. The weight of scientometric parameters ranges between 20% (QS) and 75% (USNews). The most important parameters defining ranking positions include citations, international reputation, and the number of researchers, but the correlation strength varies among ranking systems. The absolute number of publications and citations are particularly important in ARWU and USNews rankings, and scientific category normalized (field weighted) citation impact is central in THE and USNews rankings. Our results confirm that universities having outstanding results in rankings using size-independent indicators (QS and THE) compared to others have significantly lower number of students. High impact research can improve position in ARWU and USNews ranking lists. Regarding to different disciplines, the main results show that outstanding universities in THE ranking have higher publication activity in social sciences and universities which perform better in USNews and QS ranking have more publications in science, technology, and medicine fields and lower score in social sciences. In brief, here we present a comprehensive analysis of the correlation between scientometric parameters and university ranking positions, as well as the performance of outstanding universities and their correlation with different disciplines, to help decision makers select parameters for strengthening and to attract the interest of prospective students and their parents via a better understanding of the functions of different ranks.

## Introduction

Success of a higher education establishment can be measured by different metrics, like by the academic results of the admitted students, by the employment characteristics of graduates, by the participation of industry, or by the research output. In the case of universities, there are several metrics enabling qualitative assessment, including the oversubscribed proportion of the admissions, the admission score, the proportion of international students, the ratio of students to lecturers, the number of lecturers with PhDs, etc. In this field, before the publication of university rankings, the reputation of the university was the decisive argument for the choice. Expanding globalization created a widespread demand for higher university reputation^1^. Reputation, however, did not always prove to be appropriate, because it takes many, many years to gain and establish it. On the other hand, it is not so easy for a university with a history of hundreds of years to lose it either. According to several authors, a university's reputation is a decisive factor that increases the number of international students^2^.

As early as 1863, a research was published with the purpose to compare and analyze the German polytechnic schools—Hanover, Karlsruhe, and Zurich—for the upcoming reorganization of the Technical College in Prague^3^. Hundred years later, in 1986, the US News and Report US Colleges Ranking was published, which was the first national level comparison of universities. The first truly global international university ranking, the Shanghai Jiao Tong University's Academic Ranking of World Universities, was published in 2003. Since then, more and more university rankings have appeared, some of which have a long history and became recognized, while some others have lost attention over time.

The purpose of university rankings is to compare universities based on their merit and performance. There are as many methodologies as there are rankings, and their data collection can be based on data submitted by universities and data extracted from other sources and databases. Using these data, ranking systems aim to provide a common definition of value^4^. The range of indicators used is wide, there are metrics that depend on or are independent of the size of the university, and there are rankings where the reputation of the university is part of the method, which is assessed with the help of questionnaires. Despite their different methodologies, there are reasonable similarities between the rankings^5^, and a common feature of rankings is the use of bibliometric indicators.

Studies have been conducted to assess the effect of university rankings on the selection preferences of prospective students. A study mapped Chinese students' knowledge of university rankings using a survey of more than 900 students and found that thirty percent of the students was aware of ranking positions^6^. On the other hand, progress achieved in ranking lists had little or no effect on the students' choice of university^2^. According to others, the university rankings had an impact on the choice of university and among the indicators of the rankings, students consider those universities that refer to mentoring, faculty infrastructure, and general satisfaction of students as the most important aspects, while the characteristics that are aimed at research are less relevant to them^7^. Other studies have also shown that prospective students and their parents rely on university rankings when making decisions about higher education^8^. Based on data from a medium-sized German university, the university's place in the rankings had high importance for both international and national students, similarly to other determinants of reputation and quality^9^. A study using Google trends and QS World University Rankings found positive and significant relationship between QS ranking results and Google Search volumes for universities in the top 500^10^. Nevertheless, international students are mostly concentrated at large universities^11^.

For the determination of scientific output, a new branch of science, scientometrics, has emerged, the purpose of which is to provide an objective, qualitative picture of research output with the help of mostly quantitative indicators. The analysis of scientometric performance, an easily quantifiable output, is a key feature in each ranking. The aim of our study was to investigate the possible correlations between different scientometric variables and the results achieved by a university in each ranking and to find the common scientometric and discipline characteristics of universities that have achieved outstanding results in one ranking compared to other rankings. We also aimed to compare the importance of different parameters when determining ranking outcome. It’s important to emphasize that universities should avoid prioritizing rankings as their only objective. Instead, their focus should include enhancing teaching quality, advancing scientific research, and elevating the standard of their services. In this aspect, ranking is one of the tools at hand for monitoring relative output.

## Results

### Setting up a matched ranking for top universities

The ranking presented by the Times Higher Education (THE) magazine performs its own data collection for the included universities. It had common roots with QS ranking between 2004–2009, but in 2010 it switched to a different methodology. THE is based on 13 performance indicators grouped into five areas: teaching (30%), research (30%), citations (30%), knowledge transfer (2.5%), and international outlook (7.5%). All together 13 indicators are used, and out of these the total weight of bibliometric indicators is 38.5%. All indicators are normalized to university size or scientific area. Elsevier’s SciVal (based on Scopus data) is used as publication and citations source.

The QS—World University Ranking is published by the Quacquarelli Symonds (QS) company. Universities are ranked based on six key metrics in the ranking: academic reputation (40%), employer reputation (10%), faculty/student ratio (20%), citations per faculty (20%), international faculty ratio (5%), and international student ratio (5%). There is only one bibliometric indicator (citations per faculty—20%) which is normalized to the scientific area, and self-citations are excluded. Elsevier’s SciVal (based on Scopus data) is used as publication and citations source.

The Academic Ranking of World Universities (ARWU, also called ShanghaiRanking) was first published in 2003. Since 2009 the ARWU has been published by ShanghaiRanking Consultancy, which is a fully independent organization. It ranks the first 1000 universities, using six indicators, including quality of education (10%), quality of faculty (40%), research output (40%), and per capita performance (10%). The two normalized bibliometric indicators have a total weight of 40%, and one bibliometric related (number of highly cited researchers) indicator weights 20%. Clarivate’s InCites database (based on Web of Science Core Collection data) is used as publication and citations source.

The U.S.News Best Global Universities Ranking was launched by U.S.News & World Report in 2014. It ranks universities based on thirteen indicators, including global research reputation (12.5%), regional research reputation (12.5%), and eleven bibliometric indicators (75%). It contains size-dependent indicators, but some indicators are normalized. Clarivate’s InCites database (based on Web of Science Core Collection data) is used as publication and citations source.

After analyzing all four rankings, we got a common list with 470 universities included in at least one ranking among the top 300 universities. Of these, nine universities were found in one ranking only, 25 universities were found in two rankings, 48 universities were missing from one ranking, and 388 institutions were found in all four rankings.

### Ranking positions vs. determined parameters

**Figure 1:**
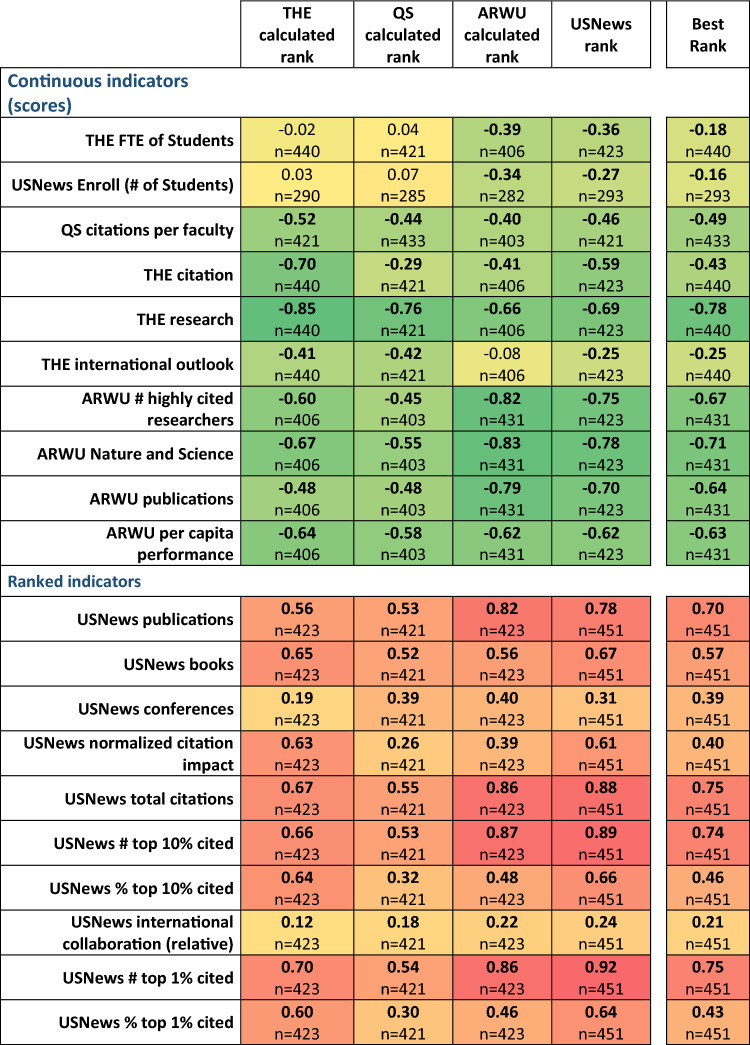
Spearman rank correlation of ranking positions and selected indicator values/ranks. High correlations show that the given indicator/rank affects the rank position in different rankings. Continuous indicators are scores, where higher values mean better positions, while USNews indicators are ranks, where the lower value means a better position. Best rank was computed by using the best positions across all four ranking for each university and reflects the power of each feature in predicting the best position of a university. Significant differences (p < 0.05) are marked with bold correlation coefficients. (The colors show the distance from the zero. Green to Yellow < 0; Yellow to Red 0 <).

First, we compared the ranking positions to the selected scientometric parameters, the number of students, and enrollment. All together 105 correlations were computed, and in 98 cases there were correlations (p < 0.05) between ranking positions and the determined parameter. The high proportion of significant correlations between ranking indicators and rank positions of the universities in most cases proves that bibliometrics related indicators play an important role in each of the examined rankings. The correlations were stronger with a particular ranking’s own indicators. We observed lower correlation in four cases: USNews international collaboration (relative) (corr.coeff = 0.24, p = 1.25e−07), USNews conferences (corr.coeff = 0.31, p = 5.86e−12), THE international outlook (corr.coeff = −0.41, p = 1.61e−19), and QS citations per faculty (corr.coeff = −0.44, p = 1.78e−22), Of these, the first three represent indicators with low weight, but the last one gives 20% weight of the entire ranking. University sizes shows medium correlation with ARWU and USNews ranking results, but there is no correlation with THE and QS rankings. The highest positive correlation coefficients were present for citations and percentage of papers in the top 1%, while the strongest negative correlations were present for two non-ranked parameters, the number of highly cited researchers and publications in Nature and Science. The complete results are presented in Fig. 1.

### Differences in ranking positions vs. determined parameters

Next, we investigated the correlation between the ranking position differences and the determined scientometric parameters. In this analysis, high correlation values mean high importance for this parameter and low correlations mean small importance in determining ranking differences between the four investigated rankings. All together 126 correlations were evaluated, and in 99 cases we found significant correlations (p < 0.05).

The most significant correlations were observed in case of the THE citations and the USNews normalized citation impact indicators. Although both of these indicators represent the citation impact of publications relative to their scientific discipline, but they use two different data sources, Web of Science and Scopus. The two indicators show higher correlation in THE–QS and in THE–ARWU pairs proving that universities with higher discipline-specific citation reach better ranking in THE compared to QS or ARWU. Albeit with lower correlation values, these universities also have better ranks in USNews. When comparing THE and USNews, THE has a positive value also linked to the higher weight of citation in THE (30%).

In case of THE–QS and of the ARWU–USNews pairs, the differences do not show correlation with university size while in case of the QS/ARWU vs THE/USNews pairs the differences show high correlation values. These results support the notion that QS and THE rankings are better for small universities with high impact while larger universities can reach better scores in ARWU and USNews rankings.

**Figure 2:**
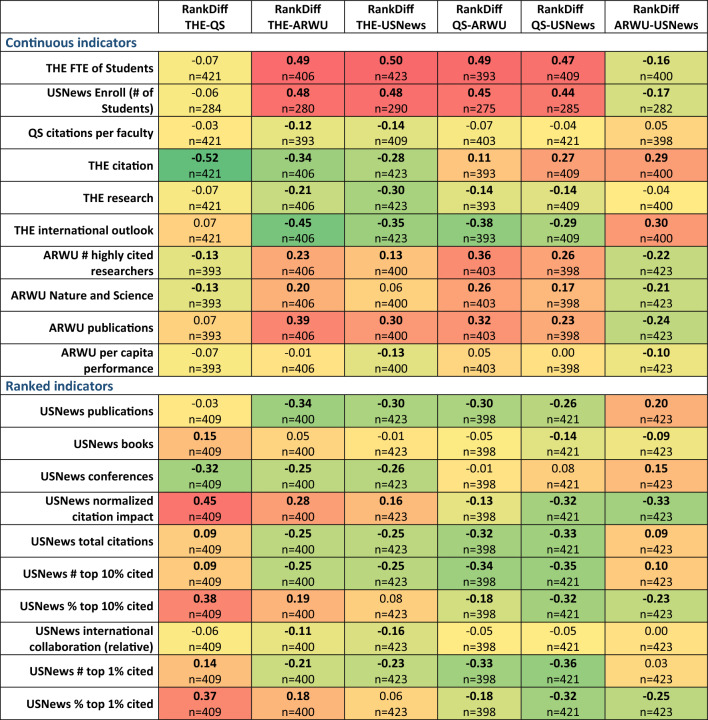
Spearman rank correlation between ranking position differences and the investigated scientometric parameters. High correlation values mean high importance of this parameter in determining the ranking-specific positions. Low correlation values mean small importance in determining ranking differences between the four investigated rankings. RankDiffs are the ranking position differences of a university between two rankings (ranked place of first ranking minus ranked place in the second ranking). RankDiff value is low (negative) if the university is ranked higher in the first ranking than in the second, and high (positive) if it is ranked higher in the second ranking. Significant differences (p < 0.05) are marked with bold correlation coefficients. (The colors show the distance from the zero. Green to Yellow < 0; Yellow to Red 0 <).

The detailed analysis results are shown in Fig. 2.

### Comparing all determined parameters to each other

We also correlated all the investigated parameters to each other across all included universities. In this, 441 associations were checked, 399 of which had a significant correlation (p < 0.05).

Particularly high correlations can be observed for USNews scientometric parameters including the number of publications in the top 10% and top 1%, and citations in the top 10% and top 1%. These results provide evidence that the overall impact of these parameters is amplified by their influence on the other parameters. Of note, the percentage of top 10% and top 1% are also positively correlated.

**Figure 3:**
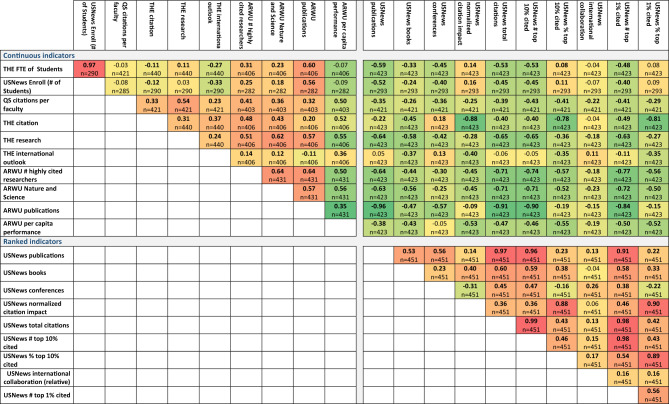
Spearman rank correlations between all the investigated parameters across all included universities. High correlations show that the given indicator/rank affects the other indicator/rank. Significant differences (p < 0.05) are marked with bold correlation coefficients. “n” means the number of universities where both indicator/rank data were available. (The colors show the distance from the zero. Green to Yellow < 0; Yellow to Red 0 <).

Similarly, significant correlations can be observed between the size-dependent parameters of ARWU and USNews. The complete results for all parameters are depicted in Fig. 3.

### Outstanding universities

**Figure 4:**
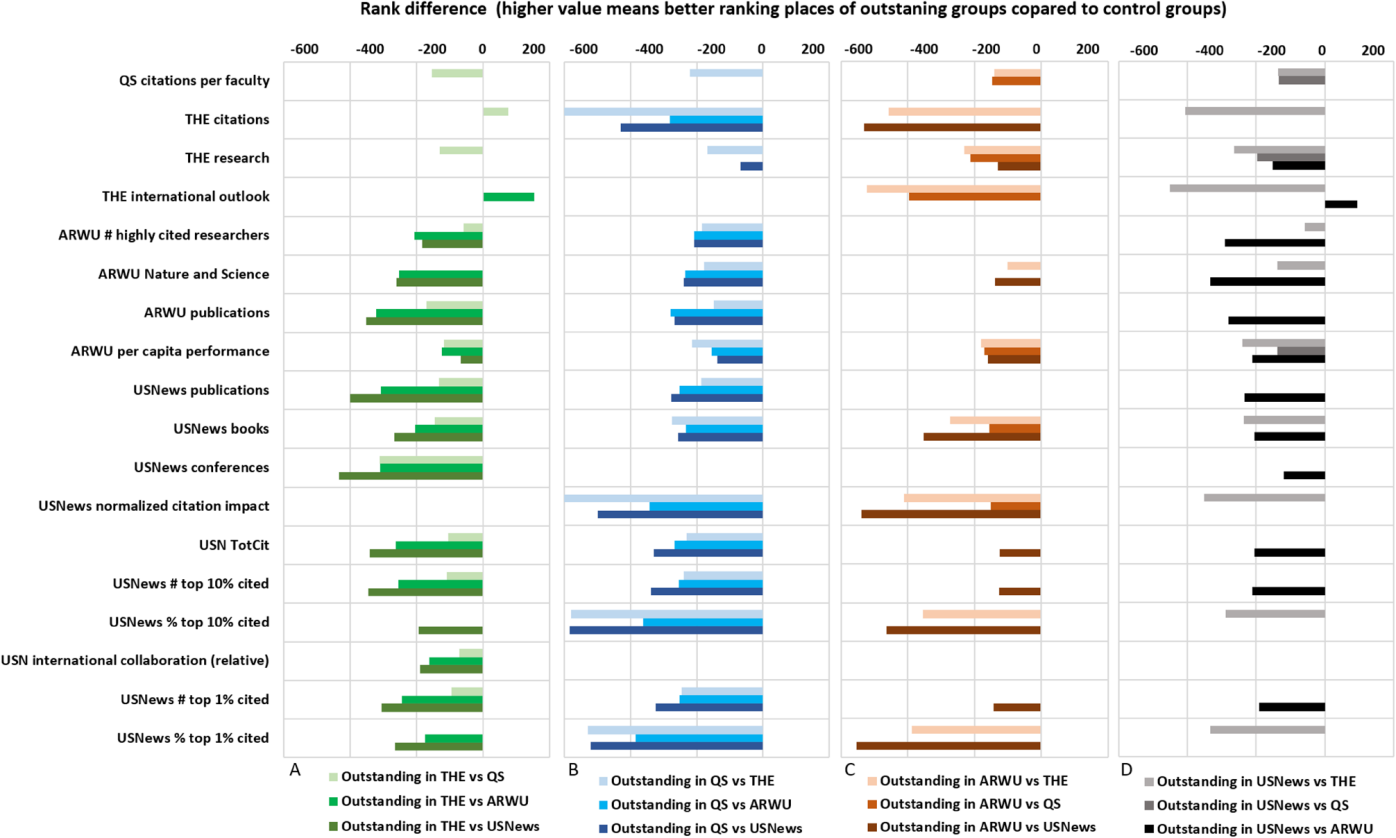
Differences of medians of indicator ranks between outstanding university groups compared to control groups comprising all non-outstanding universities in the given pair. Outstanding universities were those which had a ranking advantage of at least 100 positions compared to position in the other ranking. Outstanding universities and control groups were compared using the Mann–Whitney two-sample rank-sum test. Only those median differences are shown where the two group significantly differed (p < 0.01).

A particularly interesting analysis involves the “outstanding universities”, which have excellent position in one ranking but mediocre position in another. All together twelve outstanding university groups were compared with the control groups (which include all non-outstanding universities) for the 22 determined parameters. The analysis includes universities outstanding in THE vs. the three other rankings (Fig. 4A), universities outstanding in QS vs. the three other rankings (Fig. 4B), universities outstanding in ARWU compared to the other universities (Fig. 4C), and universities outstanding in USNews compared to other rankings (Fig. 4D). Note that because outstanding universities have better positions (lower number), the ranking difference is negative for almost each parameter.

**Figure 5:**
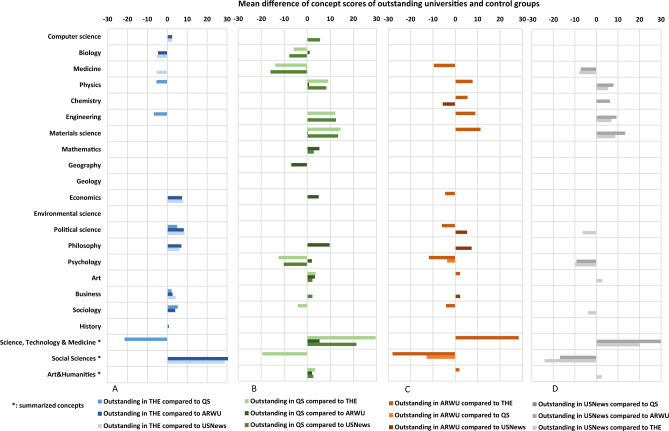
Differences of means of score values of root level concepts between outstanding university group compared to control groups comprising all non-outstanding universities in the given pair. Outstanding universities were those which had a ranking advantage of at least 100 positions compared to position in the other ranking. Outstanding universities and control groups were compared using the Mann–Whitney two-sample rank-sum test. Only those mean differences are shown where the two group significantly differed (p < 0.01).

We also studied the typical disciplines of the universities selected with this method. We examined the differences of OpenAlex root level concepts scores (19 concepts and three concept groups) between outstanding university groups and control groups. In this analysis, we found significant differences (Mann–Whitney p < 0.01, two-tailed) in case of 98 outstanding groups (see result in Fig. 5A–D). The main results show that outstanding universities in THE ranking have higher score in social sciences and universities which perform better in USNews and QS ranking have higher score in science, technology, and medicine fields and lower score in social sciences.

**Figure 6:**
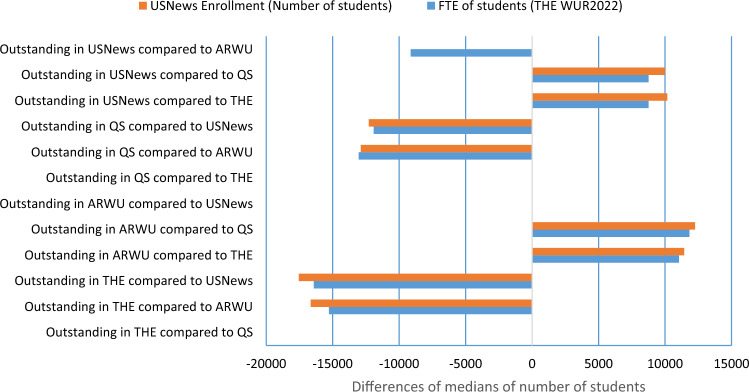
Differences of medians of university size parameters between outstanding university groups compared to control groups comprising all non-outstanding universities in the given pair. Outstanding universities were those which had a ranking advantage of at least 100 positions compared to the other ranking. Outstanding universities and control groups were compared using the Mann–Whitney two-sample rank-sum test. Only those median differences are shown where the two group significantly differed (p < 0.01).

The university size parameter was also examined when comparing outstanding and control university groups. Results show that universities outstanding in QS and THE compared to ARWU and USNews have significantly differed with lower number of students (Mann–Whitney p < 0.01 two-tailed) and conversely, universities outstanding in ARWU and USNews compared to THE and QS had higher median of number of students (Fig. 6).

## Discussion

Here, we have determined the effects of scientometric parameters in four international ranking systems. Our results confirm previous observations that there are reasonable similarities between the rankings^5^. Of course, this does not mean that all universities perform equally in each ranking—we can also confirm the size dependence, which lead to better result for larger universities^14^. Our results clearly show that smaller universities have better results in size independent rankings including the THE and QS, while bigger universities can perform better in USNews and ARWU rankings which both have size-dependent indicators. Overall, large universities can emphasize size and/or quality parameters, while smaller universities can reach better positions in staff normalized rankings.

We have determined the effect of scientometric indicators on ranking positions. Previously, it has been shown that ranking scores correlate with the publication output and citations of a university^15^. It was also established and extended that broader field coverage is also an advantage in rankings^16^. Our results confirmed that scientometric indicators play a major role in rankings. We have found significant correlations between almost all bibliometric-related indicators and positions in each examined ranking. Our results also show that the absolute number of publications and citations are particularly important in ARWU and USNews rankings, and scientific category normalized (field weighted) citation impact is important in THE and USNews rankings.

Each ranking uses different indicators to measure the performance of universities, which vary from one ranking to another. Remarkably, many of these different parameters used for the rankings are not truly independent and we found strong correlations between examined parameters in some cases. These similarities can be divided to two groups: first, the correlation is actually high because of the natural phenomenon that the indicators can be similar in different rankings, e.g., ARWU publications and USNews publications or THE citation and USNews normalized citation impact show similar data from different sources which have significant overlap (e.g., data from Web of Science or Scopus). The second group involves indicators correlated to each other within one ranking. We found that indicators measuring absolute values in USNews ranking are highly correlated with each other. For example, universities performing well in the publications indicator also rank well in the citation’s indicator, similar to the #top 1% cited and #top 10% cited indicators.

We have to mention several criticisms of university rankings formulated by different research groups. Vernon et al. summarized several doubts about the rankings, suggesting that the significance of the reputation questionnaire should be kept below 10%^12^. QS has the highest weight of reputation indicator (50%) of all the rankings we examined, followed by THE (33%) and USNews (25%). Daraio et al. abridged the main criticisms of the rankings into following four issues: monodimensionality, i.e. the rankings focus mainly on research among the education, research, and third missions of universities; statistical robustness, i.e. statistical problems of the individual indicators; dependence on university size and subject mix; and lack of consideration of the input–output structure^13^. As our results show, that ARWU and USNews rankings have high correlation with university size, while THE and QS rankings use normalized parameters.

We have to note a limitation of our study: the university ranking websites usually do not provide detailed information on the indicators, so approximations had to be used in some cases. In the THE ranking, the "Research" and "International Outlook" pillars, whose values are publicly available, were calculated by combining bibliometric and other indicators, so the effect of bibliometric parameters can be distorted in the correlation calculations. In the USNews ranking, the order of each indicator was available instead of the score values, and this contains less information. The exact ranking position in the ARWU list can only be calculated as an approximation, which also causes some bias. A second limitation is the use of different university names. In some ranking sites the English name only while in other sites the local language versions are used. We tried to identify each university in each list precisely, but we cannot completely exclude the possibility of a mismatch. Notably, as we found strong correlation between number-related parameters, it seems that at least for the THE, USNews, and QS rankings this potential bias is negligible. Finally, although we aimed to make all data openly available, the copyright of the original data sources prohibited this goal.

Unfortunately, in our study it was not possible to give a perfect guide, as one of the limitations of our project is that we only looked at bibliometric indicators (and the university's size). However, each ranking attempts to find the best universities by weighting different indicators. In our study, we found that, even when using the most objective bibliometric indicators, significant differences in the performance of several universities in each ranking can be detected when using certain types of indicators, and that publication differences in the disciplines also affect the performance in each ranking. Based on these considerations, it is likely that the use of discipline-specific rankings would be more effective than overall rankings for the study of education, research and services.

University ranking systems are on the rise and influence the perceived prestige of a university. Here, we have determined the effects of scientometric parameters on university ranking positions. Notably, there are also other reasons why one group of universities have significantly different results in rankings, e.g., diversified territorial and educational contexts can cause structural biases^17^. In this study we have identified factors significantly related to the outstanding status. Overall, strong publication activity is an important factor in each ranking, but significant differences in ranking places depend on both the selected indicators used by the ranking and the publication and citation characteristics of the universities.

## Methods

### Setting up a matched ranking for top universities

We selected four internationally recognized rankings from three continents, Europe, North-America and Asia. A common characteristic of these rankings was the availability of detailed ranking data. We used the most recent version of the four chosen rankings including the Times Higher Education World University Ranking 2022 edition (THE), the QS World University Rankings 2022 edition (QS) the ShanghaiRanking—Academic Ranking of World Universities 2021 edition (ARWU), and the U.S.News Best Global Universities Ranking 2022 edition (USNews).

### Recomputing ranking scores for scientometric indicators

Because most ranking websites do not publish exact ranking results, only bins, we had to calculate the ranking positions of the universities according to the ranking’s published methodology. For example, in ARWU, the total scores were available only for the first 100 universities, for the other universities the total scores could not be derived from the public data, so we used estimation of the total score based on the values of each indicator using their methodology.

We selected bibliometrics-related indicator values from the four rankings, which were available on the webpage of the rankings. This includes altogether twenty indicators (one indicator from QS, three indicators from THE, four indicators from ARWU, and 12 indicators from USNews) which are described in detail in Table 1. Notably, the total weight of scientometric parameters is 60% in THE, 60% in ARWU, 20% in QS, and 75% in USNews.

**Table 1:** Summary of examined scientometric indicators.

Indicator abbreviation	Ranking	Indicator original name	% of overall	size dependent	Definition	Indicator origin (database)	Indicator type
THE citation	THE	Citations	30%	no	Citation ratio compared to average in same field, area, type and year	Scopus	Citation
THE research	THE	Research (Includes research productivity)	30% (6%)	no	Number of publications (article, review)/number of staff FTE	Scopus	Publication
THE international outlook	THE	International Outlook (Includes collaboration)	7.5% (2.50%)	no	% of articles	Scopus	Publication
QS citations per faculty	QS	Citations per faculty	20%	yes	The total number of citations received by all papers produced by an institution across a five-year period by the number of faculty members at that institution	Scopus	Citation
USNews publications	USNews	Publications	10%	yes	The total number of scholarly papers—reviews, articles and notes	Web of Science	Publication
USNews books	USNews	Books	2.50%	yes	The total number of books	Web of Science	Publication
USNews conferences	USNews	Conferences	2.50%	yes	The total number of conference abstracts, proceeding papers	Web of Science	Publication
USNews normalized citation impact	USNews	Normalized citation impact	10%	yes	The total number of citations per paper	Web of Science	Citation
USNews total citations	USNews	Total citations	7.50%	yes	The total number of citations	Web of Science	Citation
USNews # top 10% cited	USNews	Number of publications that are among the 10% most cited	12.50%	yes	The number of papers that have been assigned as being in the top 10% of the most highly cited papers in the world for their respective fields	Web of Science	Publication
USNews % top 10% cited	USNews	Percentage of total publications that are among the 10% most cited	10%	no	The percentage of a university's total papers that are among the top 10% of the most highly cited papers in the world—per field and publication year	Web of Science	Publication
USNews # top 1% cited	USNews	Number of highly cited papers that are among the top 1% most cited in their respective field	5%	yes	Number of highly cited papers that are among the top 1% most cited in their respective field based on the Clarivate's Essential Science Indicators™	Web of Science	Publication
USNews % top 1% cited	USNews	Percentage of total publications that are among the top 1% most highly cited papers	5%	no	The number of highly cited papers for a university divided by the total number of documents it produces	Web of Science	Publication
USNews international collaboration (relative)	USNews	International collaboration—relative to country	5%	no	The proportion of the institution's total papers that contain international co-authors divided by the proportion of internationally co-authored papers for the country that the university is in	Web of Science	Publication
ARWU Nature and Science	ARWU	N&S—Papers published in Nature and Science	20%	yes	The number of papers published in Nature and Science between 2016 and 2020. To distinguish the order of author affiliation, a weight of 100% is assigned for corresponding author affiliation, 50% for first author affiliation (second author affiliation if the first author affiliation is the same as corresponding author affiliation), 25% for the next author affiliation, and 10% for other author affiliations	Web of Science	Publication
ARWU publications	ARWU	PUB—Papers indexed in Science Citation Index-Expanded and Social Science Citation Index	20%	yes	Total number of papers indexed in Science Citation Index-Expanded and Social Science Citation Index in 2020. Only publications of 'Article' type are considered	Web of Science	Publication
ARWU per capita performance	ARWU	PCP—Per Capita Performance	10%	no	The weighted scores of the five indicators divided by the number of full-time equivalent academic staff give PCP scores. If the number of academic staff for institutions of a country cannot be obtained, the weighted scores of the above five indicators are used	Web of Science	Publication
ARWU # highly cited researchers	ARWU	HiCi—Highly Cited Researchers	20%	yes	Number of researchers in "highly cited researchers" list by Clarivate	Web of Science	Special

We also collected available data on university sizes, which was the “Number of Students” parameter from THE and “Enrollment” value from USNews. These two parameters were the same for most universities, but they originate from different data collection processes.

Notably, in the THE ranking, the value of the Research productivity and International collaboration indicators are not published, only the aggregated value of these combined with other indicators (Research and International Outlook). As USNews ranking did not publish the scores of different indicators, only by the rank by the indicator values, we calculated the ranking positions for each used indicator in order to make these indicators easily comparable where this was reasonable.

### Creating merged ranking of universities listed among the top 300 in at least one ranking

A unified ranking of universities was created by merging universities which were among the top 300 in any of selected four rankings. Different university names in different rankings have been manually identified and merged (e.g., in some cases a local name was used in one ranking and English name in another). For each university in the merged list, we collected the ranking positions and indicator scores and/or ranking position values from all four selected rankings even in case the university was not listed in the other rankings. If the university was not listed at all in a ranking, the ranking place and the determined parameter indicators were left empty. In each case, we tried to identify the reason for being excluded from a particular ranking. In some cases, we found that QS did not list some universities because they are specialized in one specific field. Some institutions were listed as a part of a larger institution in one ranking, and separately in other rankings (e.g., Indian Institute of Technology).

### Determining typical disciplines of universities

To identify the most typical scientific fields of each university we used OpenAlex database concepts which classifies scientific works using an automated algorithm^18^. OpenAlex has 19 root level concepts, and concepts score values are aggregated at the university level. We have collected the score values of root level concepts of all universities, and we summarized score values of concepts to three categories, including (1) Science, Technology, and Medicine which contains the following concepts: Computer science, Biology, Medicine, Physics, Chemistry, Engineering, Materials science, Mathematics, Geography, Geology, and Environmental science; (2) Social Sciences, which contain Economics, Political science, Philosophy, Psychology, Business, and Sociology; and (3) Arts & Humanities, including Art and History.

### Statistical analysis

We used Spearman's rank correlation coefficient to evaluate the correlation between ranking positions and determined parameters in order to assess the overall weight of the investigated parameters. Also, Spearman’s rank correlation was computed to correlate the positions in different rankings vs. the parameters, and between the determined parameters.

In a separate analysis, for each ranking pair (THE-QS, THE-USNews, THE-ARWU, QS-USNews, QS-ARWU, USNews-ARWU, and their reverse complementary pairs) universities for which the difference in ranking positions exceeded 100 were classified as “outstanding universities” in the given pair of rankings. Parameters and score values of OpenAlex concepts of outstanding groups and control groups were compared using the Mann–Whitney two-sample rank-sum test. Statistical significance was set at p < 0.01 in the study.

## Data Availability

THE World University Ranking (WUR) Ranking 2022: https://www.timeshighereducation.com/world-university-rankings/2022/world-ranking; QS WUR Ranking 2022: https://www.topuniversities.com/university-rankings/world-university-rankings/2022; Shanghai_ARWU Ranking 2021: https://www.shanghairanking.com/rankings/arwu/2021. The other datasets used and/or analyzed during the current study are available from the corresponding author upon reasonable request.

## Abbreviations

THE: Times Higher Education World University Ranking
QS: QS World University Rankings
ARWU: ShanghaiRanking-Academic Ranking of World Universities
USNews: USNews Best Global Universities Ranking
WUR: World University Ranking
FTE: Full Time Equivalent

## References

[1] Fauzi MA, Tan CNL, Daud M, Awalludin MMN (2020). University rankings: A review of methodological flaws. Iss. Educ. Res..

[2] Soysal YN, Baltaru RD, Cebolla-Boado H (2022). Meritocracy or reputation? The role of rankings in the sorting o international students across universities. Glob. Soc. Educ..

[3] Kořistka, C. *Der höhere polytechnische Unterricht in Deutschland, in der Schweiz, in Frankreich, Belgien und England*. (R. Besser, 1863).

[4] Ramirez OF (2013). World society and the university as formal organization. Sisyphus J. Educ..

[5] Aguillo IF, Bar-Ilan J, Levene M, Ortega JL (2010). Comparing university rankings. Scientometrics.

[6] Allen RM (2019). What do students know about university rankings? Testing familiarity and knowledge of global and domestic university league tables in China. Front. Educ. China.

[7] Horstschraer J (2012). University rankings in action? The importance of rankings and an excellence competition for university choice of high-ability students. Econ. Educ. Rev..

[8] Beine M, Noel R, Ragot L (2014). Determinants of the international mobility of students. Econ. Educ. Rev..

[9] Koenings F, Di Meo G, Uebelmesser S (2020). University rankings as information source: do they play a different role for domestic and international students?. Appl. Econ..

[10] Rybinski K, Wodecki A (2022). Are university ranking and popularity related? An analysis of 500 universities in Google Trends and the QS ranking in 2012–2020. J. Mark. High. Educ..

[11] Antonova NL, Purgina ES, Polyakova IG (2019). THE impact of world university rankings on BRICS students' choices of universities (the case of the Ural Federal University). Vestn. Tomsk. Gos. Univ. Filos. Sotsiologiya-Politologiya.

[12] Vernon MM, Balas EA, Momani S (2018). Are university rankings useful to improve research? A systematic review. PLoS ONE.

[13] Daraio C, Bonaccorsi A, Simar L (2015). Rankings and university performance: A conditional multidimensional approach. Eur. J. Oper. Res..

[14] Docampo D, Cram L (2015). On the effects of institutional size in university classifications: The case of the Shanghai ranking. Scientometrics.

[15] Robinson-Garcia N, Torres-Salinas D, Herrera-Viedma E, Docampo D (2019). Mining university rankings: Publication output and citation impact as their basis. Res. Eval..

[16] Li HJ, Yin ZJ (2022). Influence of publication on university ranking: Citation, collaboration, and level of interdisciplinary research. J. Libr. Inf. Sci..

[17] Bellantuono L (2022). Territorial bias in university rankings: a complex network approach. Sci. Rep..

[18] Priem, J., Piwowar, H. & Orr, R. OpenAlex: A fully-open index of scholarly works, authors, venues, institutions, and concepts. arXiv (2022). https://arxiv.org/abs/2205.01833.

